# Patient Engagement and Patient Experience Data in Regulatory Review and Health Technology Assessment: A Global Landscape Review

**DOI:** 10.1007/s43441-023-00573-7

**Published:** 2023-09-24

**Authors:** Neil Bertelsen, Lode Dewulf, Silvia Ferrè, Rebecca Vermeulen, Karlin Schroeder, Laureline Gatellier, Ify Sargeant, Daniela Luzuriaga, Hayley Chapman, Nicholas Brooke

**Affiliations:** 1 HTAi Patient & Citizen Involvement in HTA Interest Group, Neil Bertelsen Consulting, Berlin, Germany; 2 Independent Expert, Les Contamines-Montjoie, France; 3 National Kidney Foundation, New York, NY USA; 4 Roche Pharmaceuticals, Redwood City, CA USA; 5 Independent Patient Representative, Jackson Heights, NY USA; 6 National Cancer Center Japan, Tokyo, Japan; 7 NPO Japan Brain Tumor Alliance, Yokohama, Japan; 8 Twist Medical, Burlingame, CA USA; 9 Patient Focused Medicines Development (PFMD), Brussels, Belgium

**Keywords:** Patient experience data, Patient engagement, Health technology assessment, Regulatory assessment, Real-world evidence

## Abstract

**Background:**

Working with patients through meaningful patient engagement (PE) and incorporating patient experience data (PXD) is increasingly important in medicines and medical device development. However, PE in the planning, organization, generation, and interpretation of PXD within regulatory and health technology assessment (HTA) decision-making processes remains challenging. We conducted a global review of the PE and PXD landscape to identify evolving resources by geography to support and highlight the potential of integration of PE and PXD in regulatory assessment and HTA.

**Methods:**

A review of literature/public information was conducted (August 2021–January 2023), led by a multistakeholder group comprising those with lived or professional experience of PE and PXD, to identify relevant regulatory and HTA initiatives and resources reviewed and categorized by geography and focus area.

**Results:**

Overall, 53 relevant initiatives/resources were identified (global, 14; North America, 11; Europe, 11; Asia, nine; UK, six; Latin America, one; Africa, one). Most focused either on PE (49%) or PXD (28%); few (11%) mentioned both PE and PXD (as largely separate activities) or demonstrated an integration of PE and PXD (11%).

**Conclusions:**

Our analysis demonstrates increasing interest in PE, PXD, and guidance on their use individually in decision-making. However, more work is needed to offer guidance on maximizing the value of patient input into decisions by combining both PE and PXD into regulatory and HTA processes; the necessity of integrating PE in the design and interpretation of PXD programs should be highlighted. A co-created framework to achieve this integration is part of a future project.

**Supplementary Information:**

The online version contains supplementary material available at 10.1007/s43441-023-00573-7.

## Introduction

The goal of healthcare systems and practitioners has always been to deliver better health outcomes for patients. However, despite the best intentions of working to achieve this goal, healthcare has historically made decisions for patients without actively involving them (i.e., without patients co-creating and co-implementing care and research models). This omission was largely because the burden of illness used to be driven by acute diseases that required immediate care by the physician with little or no time for patient input. Twentieth century advances in medical interventions have led to the emergence of chronic diseases as the dominant cause of illness [1], a setting in which the patient has a significant role in both primary prevention and treatment, and therefore has valuable experiences and insights to share. Numerous studies have shown that partnering with patients to address their clinical health needs delivers better outcomes, including satisfaction by both patients and practitioners, and often at reduced cost to society [2–4]. The value of partnering with patients now extends beyond clinical care, into a wider range of healthcare decisions. A recent comprehensive report by the Council for International Organizations of Medical Sciences (CIOMS) highlights the importance of also involving patients systematically and meaningfully throughout medicines lifecycle and development, including in regulatory processes, and provides consolidated evidence for the benefits of patient involvement [5].

Patients and those who look after them (such as family members or friends) have unique and relevant insights into the ways that a condition and its management impact day-to-day life. As such, diverse health stakeholders are increasingly seeking to engage with the patient community for their lived experiences and preferences in terms of treatments, risks, and outcomes. According to the US Food and Drug Administration (FDA) Patient-Focused Drug Development guidance, patient engagement (PE) is defined as “activities that involve patient stakeholders sharing their experiences, perspectives, needs, and priorities that help inform FDA’s public health mission” [6]. The term patient involvement is also widely used, sometimes interchangeably with PE [5, 7] but also with a variable meaning that can encompass more than “engagement”, depending on region and stakeholders. Although the two terms may have slightly different interpretations and understanding [8, 9], they both refer to the concept of bringing patients and their experiences, insights, and perspectives into decision-making processes with the aim of ensuring that decisions are rooted in the needs and experiences of patients. Community engagement is also used as a wider term and encompasses a concept beyond the scope of this work; therefore, we have focused on patient engagement as only one part of community engagement. Regardless of the language used to define patient input, both the engagement of patients in the process (PE) and the consideration of evidence on the patient perspectives and impacts (patient experience data [frequently referred to as ‘PED’ but as ‘PXD’ in this article to differentiate from ‘PE’]) are required. The goal is to inform and drive evidence-based decision-making through the collection and consideration of patient insights provided from patient-involvement practices, alongside evidence and data that capture the real-world experiences, preferences, and needs of patients. In this paper we use the term PE to refer to not only the act of interacting with patients, but also to a more meaningful, active, long-term, two-way collaboration with patients as valued, true partners in the development of medicines and medical technologies throughout the entire product lifecycle. Our definition of patient involvement or PE describes meaningfully working with patients systematically, over a period of time with a focus on delivering better outcomes.

Health technology assessment (HTA) and regulatory practices are deliberative processes that make judgment calls on available evidence; they require different stakeholders to consider the relative importance and impact of the evidence under review. Many HTA and regulatory bodies have long recognized that patient input is needed to provide the patient context in these deliberations, and they are increasingly including patients in decision-making processes that impact medicine and technology development [10]. As such, PE in regulatory practices is not new and has been embedded in some decision-making bodies since the late 1980s, with one early example being the establishment of the FDA patient advisory committee on HIV/AIDS in 1988 [11]. While PE has been used to date, to provide the insights needed to inform regulatory and HTA deliberations, and patient groups contributed to these decisions with their own evidence, decision makers lacked a systematic framework for patient-focused evidence. The need for further well-designed, robust evidence to provide additional clarity on the patient perspective and across a wider group of diverse patients was identified as a critical gap. Moving beyond the ad hoc collection and occasional consideration of patient evidence, the term and concept of PXD as a class of evidence that needs to be considered has emerged. The FDA defines PXD as “information that captures patients’ experiences, needs and priorities related, but not limited to: (1) the symptoms of their condition and its natural history; (2) the impact of the conditions on their functioning and quality of life; (3) their experience with treatments; (4) input on which outcomes are important to them; (5) patient preferences for outcomes and treatments; and (6) the relative importance of any issue as defined by patients” [6]. In this article, which also provides an update of a previous landscape analysis [10], we examine recent developments and evolving PE and PXD resources by geography in regulatory and HTA processes.

## Methods

### Project Design and Structure

**Fig. 1:**
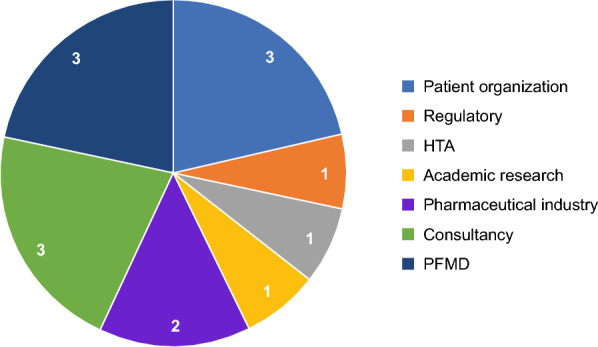
Breakdown of the 13 core contributors by stakeholder group. (Please note that one of the contributors falls under both academic research and patient organization, and so is counted twice). HTA, health technology assessment; PFMD, Patient Focused Medicines Development

The project took a phased approach, starting with the collection of information/publications on guidance, initiatives, and resources for PE and PXD in regulatory and HTA processes. Approximately 100 contributors and collaborators were approached directly through the “Patient Focused Medicines Development (PFMD) PE and PXD Project” to provide their input and signpost to potentially relevant information. This information (along with results from the literature searches described later) was then compiled into a draft report and landscape analysis (by Daniela Luzuriaga and Gary Finnegan) and disseminated to approximately 25 contributors (who were all members of the PE and PXD Project) for review and consultation. Feedback on the draft report was received from 13 core contributors who further contributed to and refined the landscape analysis. The 13 core contributors represented seven different stakeholder groups: patient representatives/patient advocacy groups, regulatory, HTA, academic research organizations, pharmaceutical industry, consultancy, and PFMD (Supplementary Table 1; Fig. 1).

Contributing members of the PE and PXD project were identified through PFMD and PFMD member networks, through participation in previous PE projects, and contribution to SYNaPsE—a platform of PE practices and initiatives. Members were invited to participate in the project and contribute to the analysis based on their expertise in PE and/or PXD, prior experience in managing, collating, developing, and using PXD, and/or their lived experience as patients. In line with good PE practices, patients were active participants in the project throughout, alongside industry and other stakeholder-group representatives. The contributors pulled in insights and examples from their networks including Health Technology Assessment International (HTAi), the Centre of Regulatory Excellence, the FDA, and the European Medicines Agency (EMA). Per International Committee of Medical Journal Editors criteria [12], core contributors were also invited to be authors of this paper.

### Identification of Global PE and PXD Resources

Google search engine was used to conduct a broad literature search (from August 2021 to January 2023) using keywords around PE and PXD, including “HTA”, “regulators”, “patient engagement”, “patient experience data”, “guidance”, “initiative”, “FDA”, and “EMA”*.* In addition, a search of “grey” literature (public information available outside of scientific or peer-reviewed journals) was conducted. This search scope included sources such as relevant research, annual reports, public hearings/conference proceedings, webinars, and data updates from the following government health authorities, professional organizations, and regulatory and HTA bodies: CIOMS, EMA, FDA, International Council for Harmonisation of Technical Requirements for Pharmaceuticals for Human Use (ICH), Medicines and Healthcare products Regulatory Agency (MHRA), Pharmaceuticals and Medical Devices Agency (PMDA), Canadian Agency for Drugs and Technologies in Health (CADTH), Center for Drug Evaluation (CDE), the Japan Agency for Medical Research and Development (AMED), Agency for Care Effectiveness (ACE), HTAi, Institute for Clinical and Economic Review, and Europe Network for Health Technology Assessment (EUnetHTA). Contributing members (see Supplementary Table 1) identified additional relevant resources and comprehensively evaluated the research.

### Assessment of the Main Focus Area of Global PE and PXD Resources

We conducted a broad thematic analysis to assess the main focus area of each resource and whether there was systematic and clear integration of PE and PXD in the approach described. Resources were reviewed for alignment with the time period of the current analysis and relevant focus on PE and PXD, and were categorized by region and stakeholder group. Information and descriptions from the title, objectives, and (where available) outputs of the resource were used to categorize its focus area as either primarily PE, primarily PXD, describing both PE and PXD but separately, or describing an integrated PE and PXD approach. Each resource was reviewed separately by a subgroup of the project team comprising four PFMD members with multiple people reviewing different resources. Where the focus area was unclear, this was discussed within the subgroup to reach consensus on thematic interpretation; categorization results were also shared with core contributors for their review and validation.

## Results

### Global PE and PXD Resources in Regulatory and HTA Processes

The landscape review and analysis provided further evidence for the growing momentum to include PE in the decision-making of global regulatory and HTA bodies with an increase in the number of relevant resources and initiatives identified compared with the previous landscape analyses (53 vs 27) [10]. The current literature search yielded a total of 63 unique PE and PXD resources (comprising initiatives, projects, and papers) in regulatory and HTA decision-making. Of these, 10 were not included in this landscape analysis following review either because they were outside of the time period for the current analysis (five resources) or because they were not relevant for the focus of PE and PXD (five resources). This report focuses on the remaining 53 initiatives and projects, of which 14 were considered global, 11 were from North America, 11 were European in origin or focus, nine were from Asia, six were from the UK, one was from Latin America, and one was from Africa (Table 1). Examples of resources that represent current PE and/or PXD initiatives in different regions are summarized in the following sections and in Table 2 [13–34].

**Table 1:** Current global patient engagement (PE) and patient experience data (PXD) resources in regulatory and health technology assessment processes

Resource/initiative	Organisation/agency	Region/country	PE focus	Integrated PE-PXD	PXD focus
1. PREFER | IMI Innovative Medicines Initiative	Innovative Medicines Initiative-PREFER (IMI PREFER)	Europe	✔		✔
2. Patient science and engagement updates from CDRH	Center for Devices and Radiological Health (CDRH)	North America—USA	✔		✔
3. Council for International Organizations of Medical Sciences (CIOMS). Patient involvement in the development, regulation and safe use of medicines	Council for International Organizations of Medical Sciences (CIOMS)	Global	✔		✔
4. FDA amplifies ‘patient science’ in product regulation	US Food and Drug Administration (FDA)	North America—USA	✔		✔
5. EUnetHTA relative effectiveness assessments: efforts to increase usability, transparency and inclusiveness	Europe Network for Health Technology Assessment (EUnetHTA)	Europe	✔		✔
6. ISPOR Webinar—differentiating between patient preferences, patient reported outcomes and patient engagement	The Professional Society for Health Economics and Outcomes Research (ISPOR)	Global	✔		✔
7. Defining patient centricity with patients for patients and caregivers: a collaborative endeavour	Article in *BMJ Innov.* (definition of PE)	Global	✔		
8. Engagement framework: EMA and patient, consumers and their organisations	European Medicines Agency (EMA)	Europe	✔		
9. The added value of patient engagement in early dialogue at EMA: scientific advice as a case study	EMA	Europe	✔		
10. 360° HTA patient involvement	Patient And Citizen Involvement group at HTAi (Health Technology Assessment International)	UK	✔		
11. Proposals for legislative changes for clinical trials	Medicines and Healthcare products Regulatory Agency (MHRA) and Health Research Authority (HRA), in collaboration with an Expert Working Group of stakeholders from across the clinical research sector, including patient representation	UK	✔		
12. Patient engagement in the design and conduct of medical device clinical studies	FDA	North America—USA	✔		
13. Stakeholder Engagement Biennial Report 2020–2021	EMA	Europe	✔		
14. Putting people first—embedding public involvement in health and social care research	National Health Service (NHS) Health Research Authority	UK	✔		
15. Guide to patient involvement in rare disease therapy development	EveryLife Foundation, Biotechnology Innovation Organisation, National Health Council, and Pharmaceutical Research and Manufacturers of America (PhRMA)	North America—USA	✔		
16. Impact of patient involvement	Canadian Agency for Drugs and Technologies in Health (CADTH)	North America—USA	✔		
17. Ministry of health labour and welfare: third term of the basic plan to promote cancer control	Ministry of Health Labour and Welfare	Asia—Japan	✔		
18. Pharmaceuticals and Medical Devices Agency—guidance on patient participation	Pharmaceuticals and Medical Devices Agency (PMDA)	Asia—Japan	✔		
19. Patient and Public Involvement (PPI) guide book	Japan Agency for Medical Research and Development (AMED)	Asia—Japan	✔		
20. 2021 Dialogue with the Agency for Care Effectiveness (ACE) Consumer Engagement and Education (CEE) Team: The Future of Patient and Public Involvement in Health Technology Assessment in Singapore	Centre of Regulatory Excellence (CoRE) at Duke-NUS Medical School, and Agency for Care Effectiveness (ACE)	Asia—Singapore	✔		
21. The Future of Patient and Public Involvement in Health Technology in Singapore	CoRE at Duke-NUS Medical School, and ACE	Asia—Singapore	✔		
22. Helping patients become involved in healthcare decision-making	ACE	Asia—Singapore	✔		
23. A framework for action to improve patient and public involvement in health technology assessment	Article—health technology assessment (HTA)	Latin America—Brazil	✔		
24. EMA Patients' and Consumers' Working Party (PCWP) Initiative	EMA	Europe	✔		
25. EMA Public Hearings	EMA	Europe	✔		
26. HTA—how patient involvement is making a difference in HTA perspectives of impact Webinar	HTAi Interest Group for Patient and Citizen Involvement in HTA (PCIG)	Global	✔		
27. Proposed ICH Guideline Work to Advance Patient Focused Drug Development	International Council for Harmonisation of Technical Requirements for Pharmaceuticals for Human Use (ICH)	Global	✔		
28. The road to treating chronic active Epstein-Barr virus infection in collaboration with citizens	AMED	Asia—Japan	✔		
29. Patients’ voices shape the drug development process through a preference survey	Pfizer	North America—USA	✔		
30. Patient involvement in health technology assessment agencies: a systematic literature review by alira health	Alira Health—HTA literature review	Global	✔		
31. Regulatory Affairs Professionals Society (RAPS)—FDA official: patients play an increasing role in rare disease drug development	Article—FDA	North America—USA	✔		
32. African Medicines Agency (AMA)	African Medicines Agency (AMA)	Africa	✔		
33. Patient Focused Medicines Development (PFMD)—PE and PXD project	Patient Focused Medicines Development (PFMD)	Global		✔	
34. Highlighting recent trends in the fast-evolving patient engagement and patient experience data landscape	PFMD	Global		✔	
35. Building from patient experiences to deliver patient-focused healthcare systems in collaboration with patients: a call to action	Article—PFMD	Global		✔	
36. The fusion of patient engagement and patient experience data: strengthening global focus and stakeholder convergence	PFMD	Global		✔	
37. PEOF: the fusion of patient engagement and patient experience data	PFMD	Global		✔	
38. “You cannot have patient experience data if patient engagement has not taken place”	PFMD	Global		✔	
39. FDA patient focused drug development glossary	FDA	North America—USA			✔
40. Qualification opinion of IMI PREFER	EMA—Qualification Opinion of IMI PREFER	Europe			✔
41. Principles for selecting, developing, modifying, and adapting patient-reported outcome instruments for use in medical device evaluation	FDA	North America—USA			✔
42. European Collaboration between regulators and healthtechnology assessment bodies	EMA and Europe Network for Health Technology Assessment (EUnetHTA)	Europe			✔
43. EUnetHTA 21	EUnetHTA	Europe			✔
44. ICMRA statement on international collaboration to enable real-world evidence (RWE) for regulatory decision making	International Coalition of Medicines Regulatory Authorities (ICMRA)—EMA, Health Canada, and FDA	Global			✔
45. EMA multi-stakeholder workshop: patient experience data in medicines development and regulatory decision-making	EMA	Europe			✔
46. NICE real-world evidence framework	National Institute for Health and Care Excellence (NICE)	UK			✔
47. Patient Involvement Strategy 2021–2025	MHRA	UK			✔
48. Inclusion of patient-reported outcome instruments in US FDA medical device marketing authorizations	Article—FDA	North America—USA			✔
49. CDE guiding principles for the application of patient report outcomes in drug clinical development (trial)	Center for Drug Evaluation (CDE)	Asia—China			✔
50. The Center for Drug Evaluation’s (CDE) draft PRO guidance	CDE	Asia—China			✔
51. The global patient experience data navigator	PFMD	Global			✔
52. Guidance 2—patient-focused drug development: methods to identify what Is important to patients—guidance for industry, food and drug administration staff, and other stakeholders	FDA (US Department of Health and Human Services Food and Drug Administration), Center for Drug Evaluation and Research (CDER), and Center for Biologics Evaluation and Research (CBER)	North America—USA			✔
53. NICE real-world evidence framework: rationale and background	NICE	UK			✔

**Table 2:** Examples of patient engagement (PE) and patient experience data (PXD) initiatives in different regions

Initiative	Purpose/summary
Global	
International Coalition of Medicines Regulatory Authorities (ICMRA) Statement	Identified opportunities for regulatory authorities to collaborate in considering how real-world evidence (RWE) can inform regulatory decision-making Areas recognized were (1) harmonization of terminology to clearly define real-world data and RWE; (2) convergence on guidance and best practice, including common principles on data quality, identification of situations where RWE can appropriately contribute to regulatory decisions, and templates for study protocols and reports that can be used by multiple authorities; (3) rapid creation of international expert groups and collaboration on processes; and (4) definition of common principles for study registration and publication of results in registries and open-source, peer-reviewed journals [13]
Proposed International Council for Harmonisation of Technical Requirements for Pharmaceuticals for Human Use (ICH) Guideline	Proposed ICH Guideline *Work to Advance Patient Focused Drug Development* identifies areas where including the patient perspective would improve “the quality, relevance, safety and efficiency of drug development and inform regulatory decision-making” Presents opportunities for global harmonization of how the patient perspective is included using a robust methodology that is appropriate for both regulated industry and regulatory authorities [14]
Global Patient Experience Data Navigator	Co-created by Patient Focused Medicines Development (PFMD) as a tool to help navigate the PXD landscape and to add clarity and structure to understanding the generation, use, and interpretation of PXD Includes tools to identify PXD use by different stakeholders throughout the product development cycle and healthcare process, determine what impacts matter most to patients and families, and select appropriate measurement methods of outcomes that matter most to patients and families [15]
Europe	
European Medicines Agency (EMA) Engagement Framework	Supporting access to individual patient’s experiences of living with a condition, its management and use of medicines Promoting the generation, collection, and use of evidence-based PXD for benefit-risk decision-making Developed by the Patients and Consumers Working Party (PCWP)—an organization that also informs global guidance provided by ICH and the Council for International Organizations of Medical Sciences (CIOMS) Patients' and Consumers' Working Party (PCWP) members come from patient organizations, industry, regulators, academia, and the World Medical Association and work collectively to highlight pragmatic points around patient involvement strategies [16]
Health Technology Assessment International (HTAi)	360° HTA Patient Involvement Project (supported by HTAi, the European Patient Academy on Therapeutic Innovation [EUPATI] and the European Patients’ Forum [EPF]) Aims to understand how methods and processes for patient involvement in health technology assessment (HTA) processes are perceived, if all stakeholders feel that patients are sufficiently involved, and to provide advice for future directions [17]
Innovative Medicines Initiative-PREFER (IMI-PREFER)	Framework that provides suggestions on how patients’ perspectives could be measured through patient preference studies and then incorporated into regulatory decision processes Framework has three main sections: (1) defining preference study aims and objectives; (2) study planning, design, and conduct; and (3) interpretation and application of study results [18]
Europe Network for Health Technology Assessment (EUnetHTA)	Joint work plan to help build a European network of experts on patient-reported outcomes (PROs), contribute to guideline development and a workshop on PXD for multistakeholders, as well as following up on areas of action identified in the ICH reflection paper on patient-focused drug development [14] The plan will further describe best practice for issues such as compensation for expert participation and, through educating experts on the difference between HTA and regulatory processes, will provide direction on how to incorporate expert input into the regulatory and HTA outputs [19]
Medicines and Healthcare products Regulatory Agency (MHRA) Patient Involvement Strategy 2021–2025	Sets out objectives to engage the public and patients during all stages of the regulatory process, and to change the internal culture of the agency such that “every member of staff considers the patient and public perspective in their decisions” Sets out objectives for multistakeholder partnerships in acknowledgement that benefits exist from sharing data and avoiding duplication of time and effort [20]
Centre for Research in Public Health and Community Care (CRIPACC)	Provides practical advice to health researchers on giving feedback to patient and public contributors, including thanking contributors and providing detailed feedback (such as impact of patient contribution and study progress) to increase motivation, confidence, learning and development, accountability, and transparency [21]
National Institute for Health and Care Excellence (NICE) RWE Framework	RWE framework is primarily aimed at pharmaceutical and health technology companies developing evidence to inform NICE guidance but is also relevant to patients and organizations that gather data and review evidence During development of the framework, NICE sought feedback through workshops and public consultation from numerous bodies, including patients and patient organizations, and revised the framework accordingly A key recommendation is that data should be collected in a patient-centered way that also minimizes the burden on patients and healthcare professionals [22]
United States	
Patient Focused Drug Development (PFDD) Guidance	Guidance published in 2020, addressing collection of patient input including sampling methods and target population definition Guidance issued in February 2022, focused on methods used to identify what is important to patients in relation to the burden of their disease and its treatment. It described best practice in conducting qualitative, quantitative, and mixed methods research, as well as considerations for the use of social media Draft guidance issued in June 2022 to help clinical trial sponsors use high quality measures of trial outcomes that are important to patients. Described how to choose, modify, or develop and validate clinical outcome assessments, including PROs, observer-reported outcomes, clinician-reported outcomes, and performance-based outcomes [23–26] The FDA's Center for Biologics Evaluation and Research (CBER) supports the PFDD mission and has a patient engagement program to incorporate patient input in their work. Initiatives include CBER’s Science of Patient Input (SPI) initiative and their Rare Disease program [27]
Medical Device Innovation Consortium (MDIC) Patient and Public Involvement (PPI) Clinical Trial Framework	Describes considerations for regulators wanting to include PPI in clinical trial design Includes leveraging existing opportunities to incorporate PPI into regulatory decision-making; identifying novel endpoints for patient preference studies and aligning these with traditional endpoints; ensuring that the PPI used is relevant to the intended patient population; and using relevant statistical methods for the trial population [28]
Patient Insights Database	Aims to support early inclusion of patients in clinical trial design Generated through patient interviews and testimonials, RWE studies, and health-related quality of life (HRQoL) research Used to further understanding of symptoms and diagnosis, treatment, HRQoL, economic burden, and patients’ hopes for new treatments [29]
US Food and Drug Administration’s (FDA) Center for Devices and Radiological Health	*Patient Engagement in the Design and Conduct of Medical Device Clinical Studies*—provides guidance on PE in medical device studies and is intended to help sponsors understand how they can use PXD to improve clinical studies for medical devices, to highlight the benefits of early patient engagement, to clarify what are considered relevant engagement activities, and to address questions/misconceptions about PE data collection for medical device design and clinical studies [30] *Principles for Selecting, Developing, Modifying, and Adapting Patient-Reported Outcome Instruments for Use in Medical Device Evaluation*—presents concepts to consider when using PRO instruments, provides recommendations around ensuring the chosen PRO instruments are sufficient for the task, and describes best practices to develop, modify, or adapt PRO instruments for an optimized outcome [31]
Japan, China, and Singapore	
Pharmaceuticals and Medical Device Agency (PMDA) in Japan	Published a report on patient participation in 2021 with the dual aim of gathering patient input and increasing awareness amongst patients of the agency’s work Made a commitment to include PROs in all medicine and device evaluations [32]
Center for Drug Evaluation (CDE) in China	Published *Guiding Principles for the Application of Patient Reported Outcomes in Drug Clinical Research and Development (Trial)* [33]
Consumer Engagement and Education (CEE) Agency in Singapore	Provided a forum for patient and volunteer organizations to publicize their work on patient and public involvement Insights included the value of patient involvement in HTA through the provision of unique first-hand experience; the importance of mutual trust, cooperation, and maintaining a two-way conversation with patients and HTAs; the need to improve patient health literacy and clarity of communication using plain language; and the importance of patient insights for rare diseases to fill data gaps [34]

### Initiatives in Europe

In the European Union, the EMA continues to make patient involvement and the collection of PXD a priority [35], including through the admission of patients as members of the EMA board and the decision to regularly invite patients to attend meetings and public hearings [36]. In addition, the EMA Engagement Framework provides a platform and roadmap for EMA’s engagement with patients and other stakeholders (Table 2) [16, 37–39]. HTAi has established several initiatives to explore optimization of patient ideas in terms of effectively incorporating them into decision-making, and to better understand the impact of patient input in terms of different stakeholder perspectives (Table 2) [17]. The Innovative Medicines Initiative-PREFER (IMI-PREFER) has produced a framework outlining how patient perspectives could be measured through patient preference studies and incorporated into regulatory decision-making [18]. The EMA acknowledged this as an important reference document but noted that there were some limitations in providing a concrete framework due to the limited experience with patient preference studies [40].

Schroeder and colleagues stressed the importance of multistakeholder collaboration in PXD approaches [41] and global health agencies have several ongoing initiatives to promote stakeholder collaboration in the use and generation of PE and PXD. In Europe, the EUnetHTA works toward building collaborations between various HTA agencies, and in September 2021, a 2-year service contract was signed by the European Health and Digital Executive Agency for the “Provision of Joint Health Technology Assessment (HTA) Work Supporting the Continuation of EU Cooperation on HTA”[19, 42]. This joint work plan has identified several areas of focus (including methodological deliverables and guidance for stakeholder interactions), and recognizes that implementation needs to be flexible, to support work towards a legislative framework on European HTA cooperation (Table 2). EUnetHTA has also developed 43 relative effectiveness assessments with an emphasis on patient involvement. The assessments are considered generalizable across countries, aiming to reduce duplication in HTA production [43]. However, it has been noted that timely patient involvement remains to be addressed along with guidance on how to demonstrate visibility of patient contribution [44].

In the UK, the MHRA aims to make consideration of patient perspectives a mandatory part of all clinical trials, and their Patient Involvement Strategy also establishes objectives for multistakeholder partnerships [20]. The UK’s Centre for Research in Public Health and Community Care provides advice to health researchers on giving feedback to patient and public contributors [21]. The National Institute for Health and Care Excellence (NICE) in England and Wales uses real-world evidence (RWE) including patient experiences to inform their guidelines and health technology appraisals, and it recognizes that such data could be used more routinely than currently to address gaps in evidence and speed up patient access to health interventions [22]. NICE therefore launched a framework in June 2022 to identify when RWE can be used to reduce uncertainties and improve NICE guidance, and to describe best practices for organizations to plan, conduct, and report studies (Table 2) [22].

### Initiatives in North America and Brazil

In the United States, the FDA has published a series of detailed guidance documents as part of their Patient Focused Drug Development (PFDD) initiatives to help stakeholders collect comprehensive and representative data and answer the question of what matters most to patients [23–26]. In a study conducted by the Medical Device Innovation Consortium, a working group of 17 experts reviewed a series of case studies to help identify the best practice for using patient and public involvement (PPI) to inform clinical trials [45]. The study led to the development of a PPI-Clinical Trial Framework, which identified several considerations for regulators wanting to include PPI in clinical trial design (Table 2). The Patient Insights Database also aims to support early inclusion of patients in clinical trial design and resultant decision-making [29]. Strides in PE have been made in the rare disease sector, where patient populations are small but often highly motivated. Working alongside patients who suffer complex and rare diseases can be crucial to finding treatments and for disease management [46]. Nicod and colleagues have provided recommendations on appropriate use of patient-reported outcomes (PROs) and health state utility values in HTA decisions for rare diseases [47].

Ways to incorporate patient feedback and input into medical technology and devices are also being explored. For example, the FDA’s Center for Devices and Radiological Health released two guidance documents in 2022 on the use of PE in medical device development (Table 2) [30, 31]. Canada’s Drug and Health Technology Agency (CADTH) have included ‘partnerships’ with patient communities (including individual patients, their families and caregivers, and patient representatives) and a pledge to work together ‘to improve and strengthen the quality and significance’ of their work, as a key element of their 2022–2025 strategic plan [48]. In Brazil, a legislative mandate for social participation makes public involvement in HTA processes compulsory. Recommendations have been made for a more systematic approach through “expansion of communication, capacity building, and transparency”, to maximize benefit from such participation [49].

### Initiatives in Japan, China, and Singapore

PPI in clinical trials/research in Japan is a relatively new endeavour [50], and until recently, examples of PPI in clinical trials were isolated cases [51, 52]. The starting point of national initiatives was a project led by the Japan Medical Agency and patient groups and supported by the Ministry of Health Labour and Welfare (MHLW) and AMED through a national survey held in 2017. The outcome of this survey, the PPI Guidebook, was published in 2019 in Japanese [53], and in 2022 in English [51]. The Ministry of Health Labour and Welfare has also emphasized the necessity of involving trained patients from the planning phase of clinical trials and in proposing patient-oriented outcomes [54, 55]. In addition to AMED, the PMDA in Japan has published a guidebook for patient centricity and is advancing PE in several sectors [32]; both organizations are beginning to collaborate with other stakeholders. China has also published guidelines around a patient-centred approach to drug development using PROs [33, 56]. With the aim of boosting patient participation in HTAs, Singapore set up the Consumer Engagement and Education agency, and in November 2021 a special dialogue was held for patient and volunteer organizations to publicize their work on PPI (Table 2) [34, 57, 58].

### Initiatives in Africa

The African Medicines Agency (AMA)—the second African health agency to help regulate medical products—was recently established to “enhance regulatory oversight and facilitate access to safe and affordable medicines across the continent” [59, 60]. The agency will also focus on harmonizing current regulatory policies/standards and will provide scientific guidelines. Although this is not directly a patient engagement or PXD resource, it does signal a move towards a regulatory system built for and by African people themselves and will promote domestic production and development of medicines within Africa. In turn, this will provide opportunities for direct patient engagement locally, along with initiatives and development of medicines directed towards patients of African descent.

### Emerging Global Initiatives

Clinical RWE and the incorporation of PE into the design of RWE studies are areas where PE/PXD is still limited; however, other regulatory authorities (in addition to NICE in the UK) are considering how RWE can inform regulatory decision-making. Following a workshop in June 2022, the International Coalition of Medicines Regulatory Authorities issued a statement identifying opportunities for regulatory authorities to collaborate in this area [13]. PFMD has co-created a Global Patient Experience Data Navigator tool to help navigate the increasingly complex PXD landscape and to add clarity and structure to understanding the “what, how, when, who, and why” of PXD. The Navigator has been designed to be relevant for diverse stakeholders and can be used to help ensure that PXD focuses on impacts that have been identified by patients as most important and meaningful, reviews available PXD measurement tools and methodologies, identifies which stakeholders are using PXD and how it is being used, and understands the impact of PXD use on decision-making [15].

### Impact of Patient Contributions on Decision-Making

**Fig. 2:**
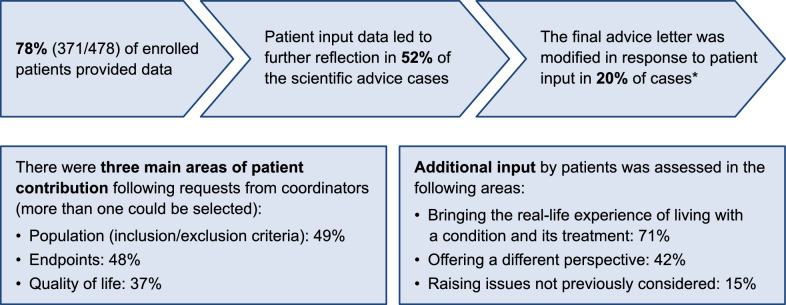
Case study of patient contributions to scientific advice provided by the EMA. The number of patient contributions and how they contributed to various aspects of scientific advice provided by the EMA was assessed as part of a 4-year case study. *In most cases where patient input did not make a change, most of the patients agreed with the development plan set out. EMA, European Medicines Agency

While there are many examples globally of patient contribution to regulatory decision-making, there is still limited documentation of the impact of such contributions [61, 62]. However, there are some insights; for example, in Europe, patient input is an established part of the medicine development process for all scientific advice provided by the EMA. A recent case study examined the extent and value of this input over a 4-year period [63]. The study quantified the number of patients involved and in which areas they contributed (Fig. 2). Importantly, patient input was shown to directly impact 52% of the cases, leading to further reflection, and in 20% of the cases, PE led to modification of the final advice letter, indicating a direct and tangible impact. The EMA also quantified the contribution of patients and healthcare professionals to their work during 2020–2021; it found that patient contributions outnumbered those of healthcare professionals across committee consultations and review of documents [64].

In their guidance for providing patient input to inform reimbursement reviews, CADTH describes the development of patient input summaries and how the patient contribution is used by their clinical and economic teams, in the CADTH clinical report, and to inform development of the review protocol [65]. The Scottish Medicines Consortium encourages patient input through its patient group submission process and has established Patient and Clinician Engagement (PACE) meetings that can be used where more detailed input is valuable, such as in the rare disease setting [66]. A summary of patient input is made publicly available, and the PACE report is incorporated in documents for committee deliberations [62, 66]. Similarly, at NICE in England, patient advocates and expert patients are able to both submit written responses and provide testimony in the deliberative committee meeting, this too is made publicly available in the HTA reports [62]. In the US since 2017, the FDA have required incorporation of a table (the “PXD Table”) within the assessment report, tabulating the patient experience data included in the submission [67]. These examples indicate that while there is increasing acknowledgment of patient input, this is currently not consistently or systematically reported, and the impact of the contribution is rarely articulated. It is likely that clearer documentation of the impact of patient contribution, with feedback to patient groups submitting their input, would demonstrate transparency in the process and also provide contributors with practical examples of what inputs are most informative [62].

### Assessment of the Main Focus Area of Global PE and PXD Resources

The collective value of PE alongside PXD is being increasingly recognized by diverse health stakeholders [41, 68–71]. There has been a call for multistakeholder collaboration and a more aligned approach to the generation, collection, and use of PXD that incorporates established elements of good practice in PE [41, 72]. The aim is to ensure that resulting insights reflect patients’ needs and priorities, meet the collective needs of diverse stakeholder groups, and minimize duplication of effort as well as burden on patients being asked for their insights [41]. We assessed whether this integrated approach is apparent in identified PE/PXD resources and found that the vast majority of resources focused either on PE or PXD; only very few had a joint focus on PE and PXD, or demonstrated an integration of PE and PXD. Of 53 resources included in this landscape review, the majority (26 [49%]) were categorized as focusing primarily on PE and 15 (28%) as focusing primarily on PXD. Overall, six (11%) resources were categorized as focusing on PE and PXD but not in an integrated approach, rather in separate sections of the resource. Only six (11%) resources were categorized as demonstrating a focus on an integrated and combined approach to PE and PXD that reflects the interdependency between the two.

## Discussion

This landscape review of recent initiatives, papers, and publications confirms and extends previous accounts of increased attention to reporting both PE and PXD by regulatory and HTA organizations. Overall, we found that this attention continues to expand globally and also to evolve from interest and openness toward guidance and expectations. The guidance and expectations that are increasingly expressed by regulatory and HTA organizations around the world pertain to both PE and PXD, albeit to varying degrees and proportions. This observation raises the question of the relationship between, and the value of, both concepts.

The FDA initiative to hold PFDD meetings [73] was in many ways a milestone in highlighting the uniqueness, value, and need for PXD as part of the regulatory review process and to complement and augment insights gained from scientific and medical data. The success of the PFDD meetings in raising awareness of the importance of PXD was in no small part due to meetings being based on quality engagement with patients testifying as an essential and early step in the process, thus providing a strong example of the value of integrating PXD and PE. Our landscape review indicates that much effort has been invested in PE, which is the focus of many initiatives. Much effort has also been invested in clarifying the format, quality, and use of PXD. However, these are largely separate approaches where the patient often still is simply the data “source” for PXD; they lack the critical integration of PE into the design, generation, collection, interpretation and use of PXD. By focusing preferentially or almost exclusively on the “data” in PXD, there is the risk of repeating the historic omission of the “patient” in PXD, and thus potentially jeopardizing the quality, value, and usefulness of the resulting PXD. Quality PE is necessary for the generation of quality PXD, and in fact well beyond that stage. PE also has an important role in the interpretation and understanding of PXD (it can uniquely help to answer the “why” question and thus explain the relevance of the observed PXD) and in helping all stakeholders understand the evidence and agree on a course of action. PXD and PE are interdependent, and both are essential for better regulatory and HTA decision-making. However, our landscape analysis highlights that there are few resources or initiatives where this interdependence is highlighted and where both are optimally integrated or even recommended.

Health stakeholders are calling on regulators and HTA bodies to give definitive guidance on how and when to conduct PE and PXD, but these agencies cannot provide robust guidance without first gaining their own experience on how PXD insights can be combined with PE to improve the quality of their deliberative processes. While collaborative global efforts such as PFMD’s Global Patient Experience Data Navigator [15] aim to provide clarity and direction around PXD, there is still a long way to go toward robust guidance.

The collective value of PXD to diverse health stakeholders has been described [41]. This indicates that development of a collaborative framework to accelerate the learnings from ongoing PE/PXD initiatives would be beneficial, as proposed at a multistakeholder forum in 2022 [74]; for example, it could provide advice and best practice examples on the generation of PXD with PE and the use of PXD and PE in decision-making. We acknowledge that there may be some limitations to this landscape review in terms of researching and providing an overview of all relevant global initiatives. However, as there is not one global database to search for such initiatives, in order to find relevant programs, in addition to relevant literature searches, we drew on expertise globally through a diverse group of stakeholders whose collective aim is to widen the reach of their connections to maximise communication and patient engagement. So, to the best of our knowledge, we have included all available resources at the time of writing but would also like to emphasize that the onus is on everyone to connect, join larger networks and to share learnings and best practice, to prevent duplication of efforts and to maximise outcomes for patients. We appreciate there is also value in systematic monitoring of the rapidly evolving and maturing PE and PXD landscape to identify and share emerging trends and positive initiatives, resources, and tools. As such, the authors recommend and plan a regular evaluation and update of this PE/PXD landscape review and invite all stakeholders globally to join this collaborative effort and to share their resources and insights. At the same time, we recognize that major barriers stand in the way of a true global collaboration and that while we extend this invite, we recognize that an active approach to seeking out more diverse collaborators is needed and will collectively be working towards ways to address this.

We also acknowledge that the review will not directly advance scientific approaches, methods, and hypotheses suitable for concretely advancing the scientific field of patient involvement, as this falls beyond the scope of a landscape review. However, by providing a comprehensive list of resources we have created a de facto global database that lays the foundation for further analysis of each resource. Specific methodologies to advance patient involvement is also the focus of ongoing work, research, and development at many of the key organisations mentioned that continue to develop resources and guidance in this area. Hence, we recommend a call to action for existing organizations and new organisations to work together to develop suitable and credible guidance. It is also important to note that whilst patient engagement is a form of community engagement, it was beyond the scope of this review to specifically address all such community initiatives, including those associated with health equity and diverse populations.

## Conclusions

Both PE and PXD are central to achieving an approach to healthcare that reflects and addresses needs, priorities, and outcomes that matter most to patients, in addition to clinical outcomes. The combination and integration of PE and PXD is needed to generate meaningful data that captures the patient experience and accurately reflects the patient perspective, and also to contextualise its interpretation within a deliberative process such as regulatory and HTA—ultimately leading to better quality (value-based) decisions. However, our landscape review demonstrates that the tendency has been to offer guidance/policies on either PE or PXD in isolation, and that the majority of approaches are not (yet) thinking about the benefits of combining the two. Although there are a few examples where PE and PXD have been integrated, such integration needs to be expanded and normalized. We are currently in a transition stage, where the supporting guidance has not kept pace and therefore, a key step forward will be developing robust guidance and evolving dynamic processes that can be adopted universally and in different contexts. For example, to recommend how and when to involve patients in the design, generation, collection, and application of PXD, and there are emerging tools that can support this integrated approach. To this end, multistakeholder co-creation of a framework for integration of PE and PXD is under way as part of PFMD’s PE and PXD project. Without this and other initiatives towards integration, we will lose key engagement opportunities and important patient influence on decision-making and slow progression towards building patient-centred healthcare systems. In addition, the PXD used by regulators and HTA are at risk of being of lower quality when collected (and interpreted) without patient engagement.

### Supplementary Information

Below is the link to the electronic supplementary material.

Supplementary file1 (DOCX 23 kb)

## Data Availability

Not applicable. All information is available in the references cited.
