# Supplementary material for: Patient Engagement and Patient Experience Data in Regulatory Review and Health Technology Assessment: A Global Landscape Review
Source: Ther Innov Regul Sci. 2023 Sep 24;58(1):63–78. doi: 10.1007/s43441-023-00573-7 (PMC10764510; doi:10.1007/s43441-023-00573-7)
Supplement: Supplementary file 1 — Supplementary file1 (DOCX 23 kb) [file 43441_2023_573_MOESM1_ESM.docx]

**Supplementary Materials**

**Supplementary Table 1** Contributing members of the PFMD patient engagement and patient experience data landscape analysis (* indicates a contributor with a global role and perspective; † indicates affiliation at time of manuscript development).

| **Contributor** | **Affiliation** | **Stakeholder group** |
| --- | --- | --- |
| Nathalie Bere† | EMA, The Netherlands | Regulatory |
| Conny Berlin* | Novartis Pharma AG, Basel, Switzerland | Industry |
| Rebecca Vermeulen* | Roche Pharmaceuticals, Redwood City, California, USA | Industry |
| Neil Bertelsen* | Health Technology Assessment International (HTAi), Patient and Citizen Involvement Interest Group, Berlin, Germany | HTA |
| Silvia Ferrè | National Kidney Foundation, New York, NY, USA | Patient organization |
| Karlin Schroeder† | Parkinson’s Foundation, New York, NY, USA | Patient organization |
| Laureline Gatellier | National Cancer Center, Tokyo, Japan (NCC)  Japan Brain Tumor Alliance, Yokohama, Japan | Academic researcher  Patient organization |
| Béatrice Serckx* | Consultant, Brussels, Belgium | Consultancy for NCC |
| Nicola Bedlington* | Millwater Partners GmbH, Vienna, Austria | Consultancy (sustainability) |
| Lode Dewulf* | Independent expert, Les Contamines-Montjoie, France | Consultancy (independent) |
| Daniela Luzuriaga* | PFMD, Brussels, Belgium | PFMD |
| Hayley Chapman* | PFMD, Brussels, Belgium | PFMD |
| Nicholas Brooke* | PFMD, Brussels, Belgium | PFMD |

HTA, health technology assessment; PFMD, Patient Focused Medicines Development.
